# Visible-Light Stiffness Patterning of GelMA Hydrogels Towards *In Vitro* Scar Tissue Models

**DOI:** 10.3389/fcell.2022.946754

**Published:** 2022-07-05

**Authors:** Anaïs E. Chalard, Alexander W. Dixon, Andrew J. Taberner, Jenny Malmström

**Affiliations:** ^1^ Department of Chemical and Materials Engineering, Faculty of Engineering, The University of Auckland, Auckland, New Zealand; ^2^ The MacDiarmid Institute for Advanced Materials and Nanotechnology, Wellington, New Zealand; ^3^ The Auckland Bioengineering Institute (ABI), The University of Auckland, Auckland, New Zealand; ^4^ Department of Engineering Science, Faculty of Engineering, The University of Auckland, Auckland, New Zealand

**Keywords:** GelMA, hydrogel, visible light crosslinking, mechanical properties, photopatterning, force indentation, projection, digital micromirror device

## Abstract

Variations in mechanical properties of the extracellular matrix occurs in various processes, such as tissue fibrosis. The impact of changes in tissue stiffness on cell behaviour are studied *in vitro* using various types of biomaterials and methods. Stiffness patterning of hydrogel scaffolds, through the use of stiffness gradients for instance, allows the modelling and studying of cellular responses to fibrotic mechanisms. Gelatine methacryloyl (GelMA) has been used extensively in tissue engineering for its inherent biocompatibility and the ability to precisely tune its mechanical properties. Visible light is now increasingly employed for crosslinking GelMA hydrogels as it enables improved cell survival when performing cell encapsulation. We report here, the photopatterning of mechanical properties of GelMA hydrogels with visible light and eosin Y as the photoinitiator using physical photomasks and projection with a digital micromirror device. Using both methods, binary hydrogels with areas of different stiffnesses and hydrogels with stiffness gradients were fabricated. Their mechanical properties were characterised using force indentation with atomic force microscopy, which showed the efficiency of both methods to spatially pattern the elastic modulus of GelMA according to the photomask or the projected pattern. Crosslinking through projection was also used to build constructs with complex shapes. Overall, this work shows the feasibility of patterning the stiffness of GelMA scaffolds, in the range from healthy to pathological stiffness, with visible light. Consequently, this method could be used to build *in vitro* models of healthy and fibrotic tissue and study the cellular behaviours involved at the interface between the two.

## 1 Introduction

The extracellular matrix (ECM) provides support for cells and is a crucial component that contributes to cell fate and behaviour due to wide variety of chemical or mechanical cues (Engler et al., 2006). Similarly, the composition and properties of the ECM is controlled by the cells that reside in it. The ECM may undergo severe changes in response to disease or to traumatic events, which can profoundly affect the integrity and functioning of the tissue. Fibrotic tissue, or scar tissue, is usually formed after an injury, but presents very different structural properties from healthy tissue. One of the main characteristics of fibrosis (or scarring) is the excess production of ECM, mainly in the form of collagen and fibronectin, by the activation of fibroblast cells into myofibroblasts (Keane et al., 2018). Consequently, the mechanical properties of fibrotic tissue are significantly modified compared to healthy tissue, and characterised by tissue stiffening. For example, atomic force microscopy (AFM) measurements on biological samples have shown that the elastic (Young’s) modulus of healthy myocardium from rats is approximately 18 kPa, whereas fibrotic myocardium can display an elastic modulus from approximately 55–800 kPa (Berry et al., 2006; Hiesinger et al., 2012). The interface between healthy and fibrotic tissues thus presents significant changes in composition and gradients of elastic modulus. Stiffness gradients play a crucial role in cell migration through what is called “durotaxis” (Isenberg et al., 2009; Sunyer et al., 2016). It is believed that durotaxis is a key factor for wound healing and scar formation, in that fibroblasts and myofibroblasts have been observed to migrate to a lesion site (Wells, 2013; Ozcelikkale et al., 2017), although there is still a debate about the occurrence of durotaxis *in vivo* (Shellard and Mayor, 2021).

Hydrogels are biomaterials that can closely replicate the structure and properties of ECM and are often used as cell culture substrates for various applications (Caliari and Burdick, 2016; Huang et al., 2017; Narasimhan et al., 2021). Durotaxis has been extensively studied *in vitro* with the help of various biomaterials and techniques to produce stiffness gradients (Whang and Kim, 2016; Xia et al., 2017; Li et al., 2021). *In vitro* fibrotic ECM models are also developed with hydrogels to replicate changes in mechanical properties and to study their influence on cell behaviour (Zhao et al., 2014; Ariyasinghe et al., 2018; Davidson et al., 2020). The influence of matrix mechanical properties in three-dimensional (3D) cultures have also been investigated (Lin et al., 2018; Crocini et al., 2020). Gelatine methacryloyl (GelMA) is a common and reliable biomaterial that enables precise control over its mechanical properties thanks to the chemical crosslinking (or curing) of the polymer network through its methacryloyl functional groups (Yue et al., 2015; Loessner et al., 2016). Gelatine on its own is perfectly biocompatible, however its melting point of 37°C and the difficulty of controlling its mechanical properties limit its application. Grafting methacryloyl groups to gelatine is a simple process that helps overcome these limitations while retaining good biocompatibility of the material.

To make hydrogels out of GelMA, the pre-polymer is mixed in solution with photoinitiators and exposed to light with a wavelength that can lead to photoinitiation, usually in the range of the absorption peak of the photoinitiator. As a result, covalent crosslinking occurs between the gelatine chains by forming bonds between the methacryloyl groups, thus reinforcing the polymer network in a controlled way through adjustment of light exposure and concentrations of the reactants. GelMA is typically crosslinked using ultraviolet (UV) light with photoinitiators, such as 2-hydroxy-1-[4-(2-hydroxyethyl) phenyl]-2-methyl-1-propanone (Irgacure 2959) (Fan et al., 2018) or 2,2′-azobis [2-methyl-N-(2-hydroxyethyl) propionamide] (VA-086) (Occhetta et al., 2015). However, when encapsulating cells in GelMA to replicate a 3D environment, Lim et al. (2019) found that UV light can be detrimental to cell survival. Visible-light crosslinking has thus been increasingly applied using other types of photoinitiators, such as lithium acylphosphinate (LAP) (Monteiro et al., 2018) or ruthenium combined with sodium persulfate (Ru/SPS) (Lim et al., 2016). Eosin Y is a readily available chemical compound that forms reactive species by activation under blue-green light (absorption peak at 515 nm) and has proven to be an efficient and safe photoinitiator to encapsulate cells inside GelMA scaffolds (Kerscher et al., 2017; Noshadi et al., 2017; Wang et al., 2018).

Several techniques can be used to create hydrogels with spatially heterogeneous mechanical properties, such as stiffness gradients. Such techniques include ascending frontal polymerisation (He et al., 2021), electrophoresis (Hu et al., 2021), drop coalescence (Lee et al., 2019), buoyancy difference of solutions (Li et al., 2019), variation of hydrogel thickness (Chin et al., 2021), or microfluidics (Lavrentieva et al., 2020). Photopatterning, or photolithography, is another method for patterning the stiffness of hydrogels by spatially varying the light irradiance. Photopatterning can be performed in a straightforward manner by using a physical photomask (Rape et al., 2015; Tomba and Villard, 2015; Kim et al., 2020; Li and Bratlie, 2021; Mgharbel et al., 2022), via direct laser writing (Song et al., 2021; Jagiełło et al., 2022), or by spatially varying the light source itself (Norris et al., 2016; Oh et al., 2022). The latter can be achieved using digital micromirror devices (DMDs), such as those contained in some video projectors. A DMD is typically an array of several hundreds of thousands to millions of micromirrors (one mirror for each pixel) that can be individually switched between two states “on” and “off”. By controlling the ratio of the on time to off time, each micromirror can produce a pixel with a specific intensity, thus creating images with various shades (Van Kessel et al., 1998). Such a system can then be used to project images onto the surface of hydrogels, thereby curing and patterning their mechanical properties (Wang et al., 2021). Photopatterning the stiffness of GelMA hydrogels with UV light has been extensively studied using some of the methods cited above (O’Connell et al., 2018; Ko et al., 2019; Li et al., 2019; Kim et al., 2020; Lavrentieva et al., 2020). To date, several studies have stiffness-patterned hydrogels through visible light (Rape et al., 2015; He et al., 2018) and used projection-based patterning of GelMA (Levato et al., 2021), but to our knowledge, this is the first study employing visible light projection-based patterning to control and pattern the stiffness of GelMA hydrogels.

In this article, we report the fabrication of stiffness-patterned GelMA constructs using visible light crosslinking with eosin Y as the photoinitiator. We synthesised GelMA from gelatine and characterised its degree of functionalisation. To obtain hydrogel stiffness in the relevant range of 10–100 kPa, to mimic healthy and fibrotic tissues, we optimised the gelation conditions by controlling the precursor concentration and exposure time. Photopatterning of GelMA was then investigated through different designs–either as distinct regions of different stiffnesses or as gradients of stiffness–and via different methods such as physical photomasks or photopatterning with a DMD. The latter also demonstrated the ability to create scaffolds with complex shapes, to demonstrate the ease by which this technique can be used to generate complex stiffness patterns to mimic tissue features, such as areas of fibrosis.

## 2 Materials and Methods

### 2.1 Materials

All the solutions were prepared with Ultrapure/Type 1 water sourced from a Milli-Q Direct 8 water purification system with a resistivity of 18.2 MΩ cm. Gelatine, methacrylic anhydride, eosin Y, triethanolamine (TEA), ninhydrin, (3-aminopropyl)triethoxysilane, 50% v/v glutaraldehyde solution, dichlorodimethylsilane, Corning sterile syringe filters and cellulose dialysis tubing were all purchased from Sigma-Aldrich (Auckland, New Zealand). Gibco phosphate buffer saline (PBS) tablets were purchased from Life Technologies (Auckland, New Zealand). *N*-vinylpyrrolidone (NVP) was purchased from Merck Pty Ltd. (Bayswater, Australia). Ethanol, acetone, and toluene (AR grade) were purchased from ECP Ltd. (Auckland, New Zealand). Polypropylene centrifuge tubes were purchased from Interlab Ltd. (Wellington, New Zealand). Silicon (Si) wafers (100 orientation, P-type, boron doped) were purchased from University Wafers (Boston, MA, United States).

### 2.2 Measurement of the Absorbance of Eosin Y

The absorbance of an eosin Y solution in PBS at a concentration of approximately 34 μM was measured between 400 and 700 nm in a UV-vis spectrophotometer (Shimadzu UV-2550, Shimadzu Scientific Instruments, Auckland, New Zealand) against a solution of pure PBS as reference. A blank transparency was also placed in between the light source and the cuvette containing the eosin Y solution to verify that the transparencies had no effect on the crosslinking experiments.

### 2.3 GelMA Synthesis

GelMA was synthesised according to Loessner et al.’s protocol (Loessner et al., 2016). Briefly, gelatine (from porcine skin, gel strength 300, Type A) was dissolved at a concentration of 10 wt% in PBS and heated at 50°C for an hour. Methacrylic anhydride (0.6 g/g of gelatine) was added dropwise to the reaction mixture, which was then left to react under stirring for 3 h. The reaction mixture was centrifuged to remove the unreacted methacrylic anhydride, and the supernatant was diluted in twice its volume of warm PBS and transferred into dialysis tubing (cellulose tubing, MWCO 12,400 kDa). The GelMA was then dialysed against Type 1 water for 7 days at 40°C. After purification, the resulting GelMA was freeze-dried and stored at −20°C.

### 2.4 Determination of the Degree of Functionalisation of GelMA

Following Zatorski et al.’s protocol, a ninhydrin assay was performed to determine the degree of functionalisation of the synthesised GelMA (Zatorski et al., 2020). As references, a 10 mg/ml gelatine solution in PBS was prepared and diluted at (90, 80, 70, 60, 50, 40, 30, 20 and 10) % in PBS. A GelMA solution in PBS at a concentration (C_nom_) of 10 mg/ml was also prepared. A ninhydrin solution in ethanol at 20 mg/ml was prepared. The gelatine or GelMA solutions were mixed with the ninhydrin solution (2.2 mg/ml final) at a 1:8 volume ratio (ninhydrin to gelatine/GelMA ratio) and heated at 70°C under stirring for 12 min. The different solutions were pipetted in triplicates into the wells of a 96-well plate, and their absorbance at 570 nm was measured at room temperature (RT). A calibration curve was plotted to represent the absorbance of the gelatine against its concentration, and a linear fit was made between concentrations of 2 and 8 mg/ml. The “apparent concentration”, C_app_, of GelMA in mg/ml was then calculated by reporting the average absorbance of the GelMA solutions to the linear fit. The fraction of available amine functions (*f*) in the GelMA can be calculated as $f=\frac{C_{app}}{C_{nom}}.$ The degree of functionalisation was then calculated as: DoF (%)=100 ×(1−f) .

### 2.5 Functionalisation of Substrates to Cast Hydrogels

Two kinds of functionalised substrates were needed to prepare the hydrogels: a glutaraldehyde-functionalised Si substrate on top of which the gel was cast, and that ensured good adhesion of the gel to the substrate, and a hydrophobic glass coverslip placed on top of the pre-gel to even the hydrogel’s surface and that allowed the light to go through it and to be easily removed.

#### 2.5.1 Glutaraldehyde-Functionalised Si Substrates

Small pieces of Si wafer (∼1 cm^2^) were cut with a glass cutter and cleaned by exposing the surfaces to UV ozone (UV Ozone Cleaner—ProCleaner, Bioforce Nanoscience, United States) for 20 min. The Si pieces were then soaked for 30 min in a 2% v/v solution of (3-aminopropyl)triethoxysilane in toluene, followed by 15 min of sonication in toluene and 10 min sonication in Type 1 water. The substrates were subsequently soaked for 30 min in a solution containing 1% v/v glutaraldehyde (from the 50% v/v stock glutaraldehyde solution) in PBS and then rinsed with Type 1 water.

#### 2.5.2 Hydrophobic Glass Coverslips

Coverslips were cleaned by sonication for 15 min in acetone and exposure to UV ozone for 20 min on both sides. Around 20 μl of dichlorodimethylsilane was then applied to each side of the clean glass coverslips and left to react for approximately 2 min. The excess silane was wiped with a paper, and the functionalised coverslips were rinsed with Type 1 water.

### 2.6 Preparation of GelMA Hydrogels

For 10 wt% GelMA hydrogels, freeze-dried GelMA was dissolved in PBS at 40°C at a concentration of 13.8 wt%, containing NVP at 3.7 wt%. A 1.66 mM eosin Y solution and a 0.67 g/ml TEA solution were prepared in PBS. For 15 wt% GelMA, a GelMA solution in PBS was prepared at 20.7 wt% containing NVP at 5.5 wt%. An eosin Y solution was prepared at 2.5 mM, and a TEA solution at 63 vol% (0.94 g of TEA dissolved in 500 μl of PBS). For both conditions, 200 μl of the pre-gel solution was prepared by mixing 15 μl of TEA solution, 45 μl of eosin Y solution and 145 μl of GelMA + NVP solution. For the 10 wt GelMA hydrogels, the resulting final composition was 10 wt% in GelMA, 0.66 mM in eosin Y, 5 wt% in TEA, and 2.67 wt% in NVP. For 15 wt% GelMA hydrogels, the final composition was 15 wt% in GelMA, 0.5 mM in eosin Y, 7.5 wt% in TEA, and 4 wt% in NVP. The pre-gel solution was sonicated for 5 min at RT to remove the dissolved O_2_ and then melted again at 40°C to enable pipetting. 9.3 μl of the pre-gel solution was pipetted in a metal ring (6 mm diameter, 210 μm thickness) mounted on a glutaraldehyde-functionalised Si substrate and then covered by a hydrophobic glass coverslip. The sample was subsequently exposed to a high-power green LED with a centre wavelength of 525 nm (Thorlabs Inc., United States, SOLIS-525C), or to the light of the projector set-up, for times between 1 and 8 min. The sample received an irradiance of approximately 160 mW/cm^2^ for the high-power LED or a maximum of approximately 128 mW/cm^2^ for the projector set-up. Irradiance was measured with an optical power meter (Newport Corporation, model 1936-C, United States). After crosslinking, the hydrogels were soaked overnight in 2 ml of PBS in the dark at RT.

### 2.7 Photomask-Based Stiffness-Patterning

#### 2.7.1 Binary Pattern

Photomasks were placed on top of the hydrophobic coverslip for stiffness-patterned hydrogels before crosslinking under the green light. The photomasks were designed on a vector graphics software (Inkscape) and subsequently printed in black and white at 1,200 dpi on transparencies with a laser printer. These photomasks are referred to as their defined percentage of opacity for the black colour in the software, i.e., a “0–50” mask displays 0 and 50% opacity areas.

#### 2.7.2 Gradient Pattern

To create gradients of stiffnesses with the green LED, we used a continuous optical density (OD) filter (Thorlabs Inc., NDL-10C-4, United States), consisting of a glass rectangle covered with a linear gradient of metal coating. The optical density of this filter ranged from 0.04 to 4.0 over a length of 50 mm. The filter was positioned on top of the mould containing the pre-gel solution before crosslinking the gel with the 525 nm LED.

#### 2.7.3 UV-Vis Spectroscopy Measurements of the Printed Photomask Transmittance

To measure the light transmittance of the printed photomasks, we printed them on transparencies with a laser printer at 1,200 dpi and then positioned them in the UV-vis spectrophotometer (Shimadzu UV-2550, Shimadzu Scientific Instruments, Auckland, New Zealand) in between the light source and the sensor. The transmittance of the photomasks was measured at 525 nm (wavelength of the green LED).

### 2.8 Projection-Based Stiffness Patterning

For projection-based stiffness-patterning, we built a system that consisted of a modified digital projector (Optoma, EX525ST) containing a digital micromirror device (DMD). The projection lens and colour wheel were removed and replaced with a lens (Nikon, 50 mm) followed by an optical filter (Thorlabs, MF510-42) with a centre wavelength of 510 nm and bandwidth of 42 nm (full-width at half-maximum). Focusing the image at the top of the hydrogel mould gave an image over an area of approximately 8 mm × 6 mm. A presentation program (Microsoft PowerPoint) was used to design the patterns projected at the surface of the pre-gel solutions. The greyscale values in the pattern were set from black (greyscale value = 0) to white (greyscale value = 1) in the software. A timer for the slide was used to set the exposure time to 4 min.

#### 2.8.1 Irradiance Measurements

Slides with a defined greyscale value were projected on the sensor of an optical power meter to measure the irradiance of the projector as a function of the defined greyscale value (Newport Corporation, model 1936-C, United States). The resulting power was measured and divided by the area of the slides on the sensor to calculate the irradiance, which was then plotted against the greyscale value.

#### 2.8.2 Sliding Mask

A simple animation of a black sliding mask (irradiance of 1.0 mW/cm^2^), revealing a white background at an irradiance of 128 mW/cm^2^, was made in Microsoft PowerPoint, and moving at a velocity of 10.7 pixel/s. The animation was then projected onto the pre-gel solution. After the sliding mask reached the other extremity of the sample, a fully white pattern was shone for 1 min to further crosslink the hydrogel.

### 2.9 Mechanical Characterisation of the Hydrogels With Atomic Force Microscopy

The elastic modulus measurements of the hydrogel samples were performed on an MFP-3D Origin AFM (Asylum Research, Santa Barbara, United States) in a liquid environment at RT. After being rinsed overnight, the 2 ml of PBS in which each hydrogel was soaking was replaced. Pre-calibrated silicon nitride cantilevers with spring constants ranging between 0.07 and 0.08 N/m with a 5 μm glass bead at the tip (Novascan, United States) were used. The spring constant of each cantilever was precisely calibrated by the manufacturer beforehand, and its value was subsequently updated in the AFM software. Before each set of measurements, force-indentation curves were measured on a clean Si substrate in PBS to calibrate the DeflInvols value. Each hydrogel-coated Si substrate was fixed on a glass slide with double-sided tape, put on the AFM stage, and a droplet of PBS was placed on top of the gel. The parameters used for measuring the elastic moduli of the hydrogels were adapted from Schillers et al. (2017): 100 force-indentation curves over an area of 20 μm × 20 μm (= 1 force map) were recorded, typically with a scan rate of 0.7 Hz, travel range of 2–4 μm, tip velocity of 3 to 6 μm/s and trigger force of 2.5–5.0 nN. At least four force maps were recorded over the sample’s area for each hydrogel. The resulting force maps were analysed in the acquisition software, Asylum Research Software AR16 (version 16.10.211), operating in Igor Pro (version 6.38, WaveMetrics, United States), and the approach section of the curve was fitted with the Hertz model (Hertz, 1882). The equation used to fit force curves to approximate the sample’s elastic modulus is the following:

$$F_{app}=\;\frac{4}{3}\frac{E_{s}}{1-\;\vartheta_{s}^{2}}R^{1/2}d^{3/2}$$

with *F*_*app*_ the applied force in N, E_s_ the sample’s modulus in Pa (N m^−2^), *ϑ*_*s*_ the sample’s Poisson’s ratio (dimensionless), R the radius of the bead in m, and d the depth of indentation in m (Matzelle et al., 2003). The value of the Poisson’s ratio of the gels was assumed to be 0.33 (Greaves et al., 2011). A fit between 10% and 90% of the maximum applied force was used to quantify the elastic modulus of the hydrogel samples from the force-indentation curves (see Supplementary Figure S1 for examples of fits on force curves). A histogram of the elastic modulus values was generated for each force map, and a Gaussian fit was applied to it. This fit provided a mean value of the elastic modulus and the standard deviation (SD) for each force map. If some force curves within the force maps displayed irregular shapes or values, these were excluded from the fit by applying a mask that excluded force curves with a fit above or below a certain threshold of elastic modulus.

For force measurements on gradient hydrogels, 100 force-indentation curves over an area of 10 μm × 10 μm were recorded every 500 μm or 1 mm, measured with the AFM stage micrometre, along the direction of the gradient. The data are presented as the mean value of the elastic modulus ±SD, as calculated by the Gaussian fit on the histogram of each force map.

### 2.10 Statistical Analysis of the Mechanical Characterisation Results

Several sets of data were used to plot the hydrogels’ elastic moduli as a function of exposure time. For each exposure time with the 15 wt% hydrogels, seven replicates were tested, and four replicates were made for the 10 wt% hydrogels. For each hydrogel, a minimum of three force maps and a maximum of six were generated over different sample regions. For each force map, the mean value of the elastic modulus and its SD, generated from the Gaussian fit of the histograms, were collected. The mean values of the elastic modulus were averaged over all the generated force maps pooled together to calculate the average elastic modulus of the hydrogels for each condition (concentration and exposure time), provided that the SD of the force map was <30% of its mean value. If the SD was >30%, the corresponding force map was excluded from the analysis. Force maps with irrelevant overall elastic modulus were also excluded from the analysis (i.e., very low elastic modulus for long exposure time). The standard error was also calculated over the corresponding force maps with a SD < 30% for each average elastic modulus. For each GelMA concentration, the resulting average elastic modulus was plotted against the hydrogels’ exposure time, and the corresponding error bars represent the standard error of the data set.

For assessment of the stiffness-patterned hydrogels with the binary masks, one control gel and two patterned replicates were tested per condition. Four force maps were made on the control gels at different places on the surface, and the mean values and SDs of the force maps were extracted. The average elastic moduli of the control gels for each condition were calculated by averaging the four mean values, and the standard errors of the data sets were also calculated. Concerning the patterned gels, for each sample, three force maps were made on the non-masked region and a minimum of four force maps were made on the masked region of the gel. Similarly, the mean values and SDs of the elastic moduli were extracted for each force map. Subsequently, the mean values per mask region were averaged over the two samples, and the standard error was calculated. The graphs represent the values of the average elastic modulus, and the error bars represent the standard error.

For assessment of the gradients of stiffness, each point on the graph represents the mean value of elastic modulus from the Gaussian fit of the histogram of a single force map. The error bars represent the SDs of the Gaussian fit.

For statistical comparison of two data sets, a Student’s *t*-test (two-tailed distribution, two-sample equal variance) was performed on the pooled mean elastic modulus values obtained with the force maps measured from each sample.

## 3 Results and Discussion

### 3.1 Synthesis of Gelatine Methacryloyl

**FIGURE 1:**
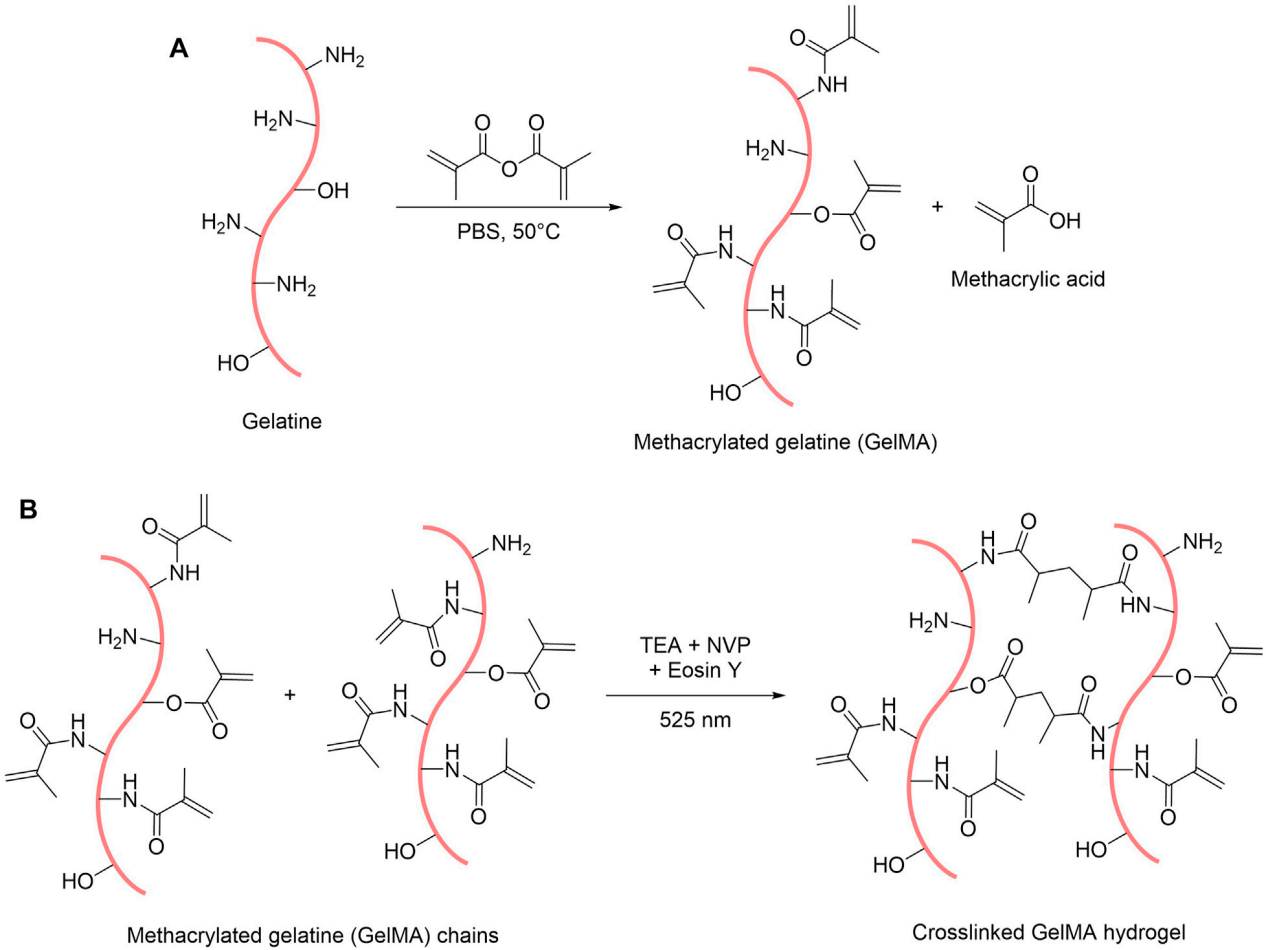
Scheme of the reactions for the synthesis of GelMA **(A)** and for the formation of GelMA hydrogels **(B)**.

Gelatine methacryloyl (GelMA) was synthesised by reacting gelatine with methacrylic anhydride in phosphate buffer saline (PBS) under stirring for approximately 3 h at 50°C. This process allows the functionalisation of the gelatine backbone by grafting methacrylamide and methacrylate groups on the free amine and alcohol functions, with methacrylamide groups being the majority of the functions (>90%) (Yue et al., 2015; Loessner et al., 2016) (Figure 1A). To determine the degree of crosslinking achievable with the synthesised compound, we assessed the degree of functionalisation (DoF) of the resulting GelMA with a ninhydrin assay (Zatorski et al., 2020). This assay relies on the reaction of ninhydrin with free primary amine functions present on the gelatine backbone. When bound to an amine, ninhydrin forms Ruhemann’s Purple, a purple-coloured soluble product whose absorbance peak is at 570 nm. It is then possible to determine the amount of free primary amines in the sample by measuring the absorbance at 570 nm of a solution containing gelatine or GelMA reacted with ninhydrin. Therefore, by comparing a sample of unreacted gelatine and a sample of GelMA, it is possible to calculate the amount of unreacted amine functions in the GelMA and thus calculate its percentage, or degree, of functionalisation. We thus estimated the DoF of our synthesised GelMA to 80%, which translates a relatively high functionalisation of the gelatine with this synthesis method (Shirahama et al., 2016). However, this assay does not take into account the free alcohol groups on the gelatine backbone, thus introducing a small error on the assessment of the DoF by ignoring the amount of methacrylate groups present in the GelMA.

GelMA was obtained as a white foam after purification by dialysis and freeze-drying. To make hydrogels out of this foam, GelMA was dissolved in PBS and mixed with three other compounds: eosin Y, which is the photoinitiator activated by blue-green light (absorbance peak at 515 nm in PBS, see Supplementary Figure S2), triethanolamine (TEA) that acts as a co-initiator to generate reactive species, and *N*-vinylpyrrolidone (NVP), which is a co-monomer that accelerates the reaction (Noshadi et al., 2017). By exposing this pre-gel mix to a green 525 nm high-power light-emitting diode (LED), the crosslinking reaction can occur and produces a hydrogel by trapping water within the polymer network (Figure 1B).

### 3.2 Optimisation of the Gelation Conditions and Mechanical Characterisation

Fibrotic, scarred, tissue is generally stiffer than healthy tissue. Therefore, it is important to optimise gelation to reach elastic modulus values for both healthy and scarred tissue. For cardiac tissues for example, the relevant range of elastic moduli from 10 to 100 kPa that mimics healthy and fibrotic cardiac tissues (Berry et al., 2006; Hiesinger et al., 2012) can be achieved by tuning several parameters of the hydrogel. Herein, we focussed on pre-polymer concentration and on exposure time to green light, which initiates the cross linking. Concerning the “photoinitiator” concentrations (the term “photoinitiators” will subsequently refer to eosin Y, TEA and NVP altogether), we chose to use final concentrations in the hydrogel of 0.66 mM in eosin Y, 5 wt% in TEA, and 2.67 wt% in NVP for a final GelMA concentration of 10 wt%. To reach the highest possible crosslinking, we also increased the GelMA and photoinitiator concentrations to 15 wt% GelMA and 0.5 mM eosin Y, 7.5 wt% TEA, and 4 wt% NVP, respectively. These concentrations were chosen as they enable the fabrication of very stiff hydrogels [up to 90 kPa of compressive modulus (Xu et al., 2021)] and can be used to encapsulate viable cells–even though it has been shown that cells encapsulated in lower GelMA concentrations display better proliferation and functionality.

**FIGURE 2:**
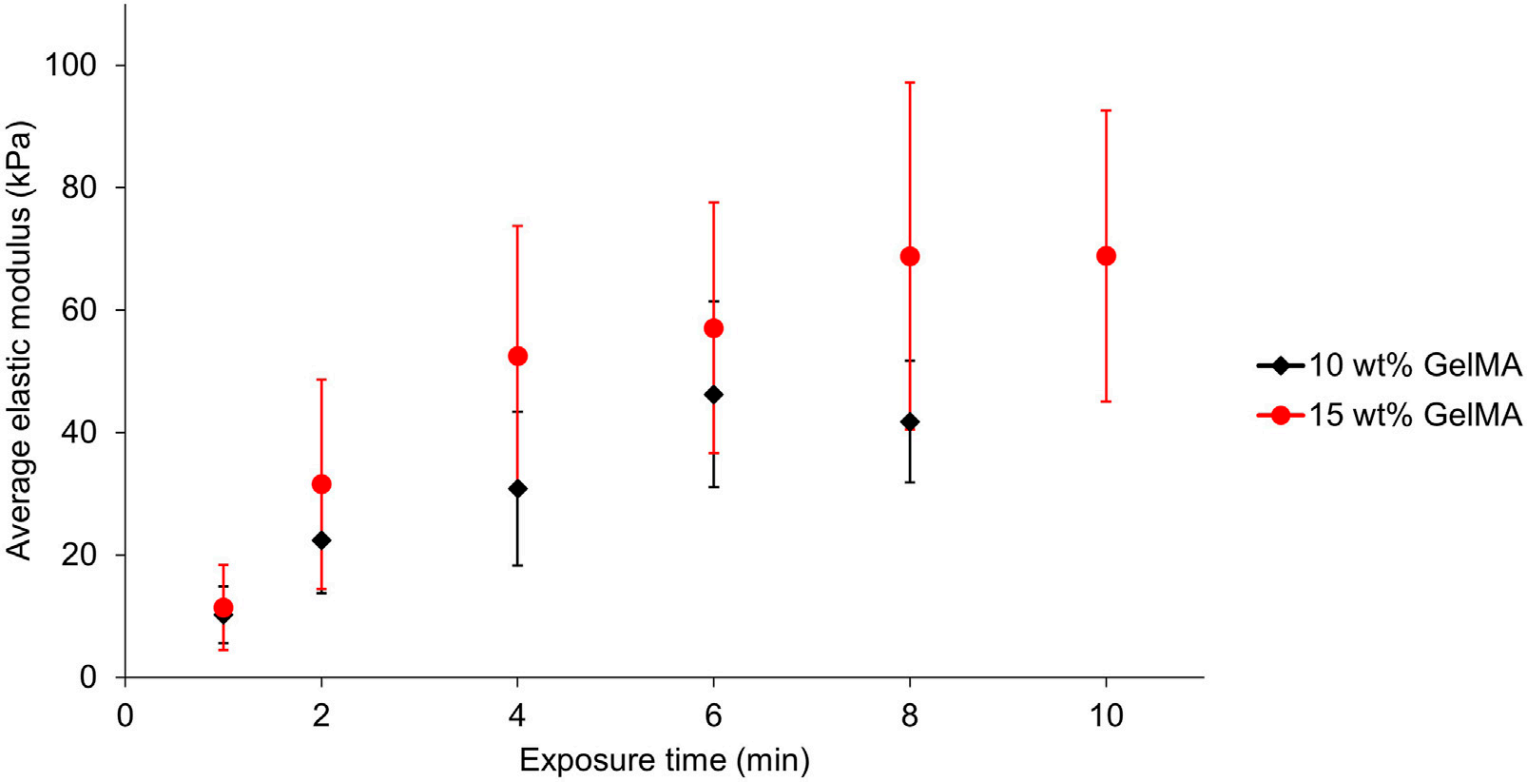
AFM force indentation measurements of the average elastic moduli of 10 wt% (black diamond) and 15 wt% (red circle) GelMA hydrogels as a function of their exposure time to the green LED. The error bars represent the standard error (10 wt%: between 15 and 21 force maps over four separate gels for each exposure time; 15 wt%: between 27 and 31 force maps over seven separate gels for each exposure time).

Force indentation measurements with atomic force microscopy (AFM) were used to assess the mechanical properties of the GelMA hydrogels (Figure 2). For this, 10 and 15 wt% GelMA pre-gel solutions were exposed to green light (LED setup as described in Section 2, irradiance of 160 mW/cm^2^) for times from 1 to 8 min, and kept in PBS at room temperature (RT) overnight. The average elastic modulus of each sample was assessed by generating 100-point force maps at least at three different locations of the samples. By fitting the force-indentation curves of the force maps with the Hertz model (Hertz, 1882), the average and standard error of the elastic moduli was obtained (Figure 2). The results showed that, by increasing the exposure time to the green LED, the average elastic modulus of the hydrogels also increased. This is expected, as exposing the pre-gel solution for a longer time to the green light increases the number of bonds formed between the gelatine chains, thus resulting in a stronger polymer network and stiffer gel. The elastic moduli obtained with these exposure times range from approximately 10–46 kPa for 10 wt% GelMA and from approximately 10–70 kPa for 15 wt% GelMA, giving ranges within the initial target range of 10–100 kPa. In both conditions, the average elastic modulus of the hydrogels stabilises after a certain exposure time (6 min for 10 wt% and 8 min for 15 wt%). This shows that, after a certain exposure time at fixed irradiance (160 mW/cm^2^), the maximum number of crosslinks between the gelatine chains has been reached, either because of the limiting amount of free methacrylate groups that could react together or because of the limiting amount of photoinitiators.

For the 10 wt% hydrogels, the average elastic modulus decreased after 6 min of crosslinking. This decrease was also observed for some sets of 15 wt% GelMA samples after 8 min (Supplementary Figure S3). A previous study from Zhu et al. (2018) mentions that this phenomenon could occur because of the photobleaching of eosin Y. Alternatively, this phenomenon could be due to thermal effects on the hydrogel due to the high power of the light that gives rise to heating in the material. Measurements of the temperature under the green LED over time showed an increase from RT to a maximum of 47°C after approximately 7 min (Supplementary Figure S4). Prolonged exposure of the surface of the gel to such temperature could induce melting of the gelatine and disorganisation of the chains resulting in a looser network and reduced elastic modulus.

The data points in Figure 2 display large error bars, which highlight the significant variation of elastic modulus between the replicates despite rigorously using the same crosslinking conditions each time. The stiffness variation within each sample is actually considerably lower (Supplementary Figure S3). A possible explanation for these variations is the time required to pipette the 9 μl into the mould and place it under the lamp before crosslinking. The pre-gel solution was heated at 40°C to keep it liquid and transfer it into the mould. However, the liquid can cool down very rapidly with such a small volume. Several studies found that cooling down a GelMA pre-gel solution to temperatures below 25°C before crosslinking actually increased the elastic modulus of the crosslinked hydrogel compared to crosslinking the pre-gel solution directly at RT (Rizwan et al., 2017; Young et al., 2020; Chansoria et al., 2021). This is due to the physical gelation of the gelatine before light-curing, which induces a better organisation of the gelatine chains resulting in a higher crosslinking density. Our significant variations in the elastic modulus may arise from the same effect due to different pipetting and sample preparation times prior to crosslinking.

We verified the influence of physical crosslinking before light curing by placing the pre-gel solutions in the moulds at 4°C for 15 min. The results showed a significant increase in elastic modulus for the gels first placed at 4°C compared to those directly crosslinked under the lamp right after pipetting into the mould (Supplementary Figure S5). Consequently, systematically placing the pre-gel solutions at 4°C before crosslinking could, at the same time, allow us to obtain reproducible hydrogels with higher elastic moduli. However, this process may not be compatible with the encapsulation of cells into the hydrogels, as exposure of several minutes to cold temperatures could potentially harm them. Pipetting the solution in the mould and placing it as fast as practically possible under the lamp was the method employed here, but variation in this amount of time (at the scale of a few tens of seconds) remained hard to avoid. Placing the substrate on a thermoelectric cooler/heater kept at 37°C during the whole process of crosslinking could also help reduce the variability in the resulting gels elastic modulus. It is also important to align the samples with the emission axis of the lamp to reduce errors due to uneven illumination. Therefore, a sample holder to guide the alignment was added and used for the 10 wt% GelMA hydrogels, likely contributing to the smaller variation seen in the stiffness for these gels (Figure 2).

### 3.3 Photomask-Based Stiffness Patterning

After confirming the ability to control GelMA hydrogels’ elastic modulus through the exposure time, the next step was to pattern regions of different stiffnesses using photomasks to spatially vary the irradiance, and thereby the crosslinking. For these experiments, gels with 10 wt% GelMA were used as this concentration is more suited for further cell culture applications (Xu et al., 2021). By designing patterns of opacity on the photomasks, it becomes possible to also pattern the stiffness. In this study, we used two types of photomasks placed between the green LED and the pre-gel solution during crosslinking. Both types of photomasks were applied to GelMA hydrogel samples, which were then characterised with force indentation measurements with AFM to confirm the patterning of the elastic modulus.

#### 3.3.1 Binary Pattern

**FIGURE 3:**
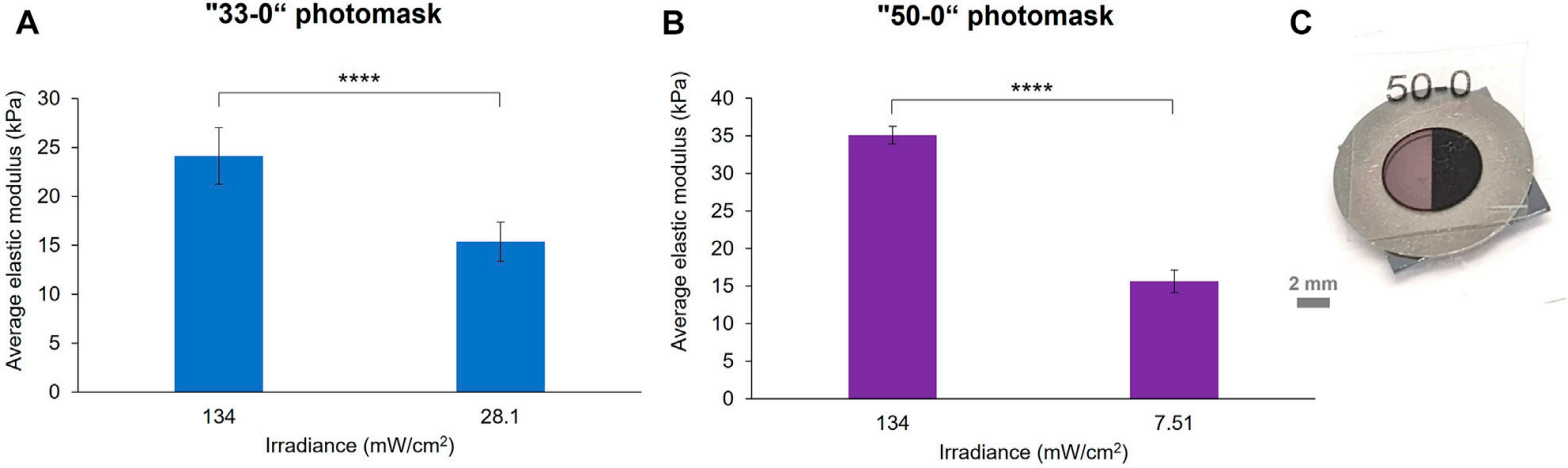
Stiffness patterning of 10 wt% GelMA hydrogels crosslinked for 4 min under the green LED with two binary photomasks: “33–0” **(A)** and “50–0” **(B)** masks. The elastic modulus of the hydrogels was significantly reduced by the opaque area of the photomask, which modulated the green LED irradiance (error bars are the standard error over at least six force maps on two samples per photomask, *p* < 0.0005). Photograph of a “50–0” binary photomask placed on top of a pre-gel solution into a mould **(C)**.

As a first simple type of patterning, binary photomasks were used (Figure 3C). These were designed on a vector graphics software (Inkscape) as disks of 6 mm in diameter, which aligned with the metal ring mould used for making the hydrogels. The disks were divided into halves: one fully transparent (0% opacity on the software) to serve as a control, the other half with a defined opacity of 33% or 50%, as defined by the software. The masks were printed at 1,200 dpi in black and white on transparencies with a laser printer and placed on top of the hydrogel samples before crosslinking. Beforehand, we ensured that the transparency did not disrupt the absorbance of eosin Y by measuring its absorbance spectrum with and without blank transparency in front of the light source (Supplementary Figure S1). The transmittance of the photomasks was also measured by UV-vis spectroscopy at 525 nm, the wavelength of the green LED, and used to calculate the resulting irradiance compared to the native one of the green LED (Supplementary Table S6). Two sets of 10 wt% GelMA hydrogels were prepared with printed photomasks placed on top of the samples during crosslinking. A crosslinking time of 4 min was chosen for these experiments, as this is in the range of the elastic modulus vs. exposure time curve (Figure 2), and because it results in a sufficiently high gel stiffness for a relatively short exposure time. Indeed, for further cell culture applications, it is better to limit the time that cells are left at RT to prevent cell death. Figure 3 shows the effect of a 33% opacity mask (Figure 3A) and a 50% opacity mask (Figure 3B) on the elastic modulus of the hydrogels according to the resulting irradiance.

For both conditions, a decrease in the average elastic modulus of the masked region was observed compared to the transparent region: from 24.12 to 15.36 kPa for the “33–0” photomask and from 35.11 to 15.66 kPa for the “50–0” photomask. It is interesting to observe that the percentage of decrease in elastic modulus between the two regions is approximately the same value as the defined opacity percentage of the photomasks: a 36% reduction in elastic modulus for the “0–33” photomask and a 55% decrease for the “0–50” photomask. However, the stiffness reductions did not correspond to the percentage of decrease in the irradiance, which was 79% for the “33–0” photomask and 94% for the “50–0” photomask. This highlights a non-linear correlation between the irradiance received by the GelMA and its resulting crosslinking ratio. A significant difference of stiffness between the 0% opacity of the two samples was noted, this stiffness variation was within the elastic modulus inter-sample variance of our GelMA system (Figure 2).

Furthermore, with this kind of photomask, we observed under the microscope a sub-patterning at the surface of the hydrogels due to the halftoning carried out in the printer to simulate greyscale tones (Supplementary Figure S7). The size of the ink dots is around 100–120 μm, and the less opaque photomask is achieved by a lower spatial density of dots, leaving transparent gaps where the crosslinking can occur at its maximum. The difference in stiffness between the area of the gel under the ink dots and the gaps was characterised, and we found a significant difference between the two: around 13 kPa under the ink dots and around 21 kPa under the transparent gaps. In fact, the results in Figure 3 were obtained by probing the gels at the positions of the ink dots. While this sub-patterning is not desired for mechanotransduction studies of cells responding to gradients or step changes in modulus, simple binary-coloured printed masks have the potential for patterning features down to the size of pixels. This kind of sub patterning in gels cured using printed photomasks has not been reported in UV cured hydrogels (Kim et al., 2020). This might be due to several effects, such as differences in light diffraction as well as reaction times (longer reaction times lead to more diffusion and blurring of pattern).

#### 3.3.2 Gradient Pattern

**FIGURE 4:**
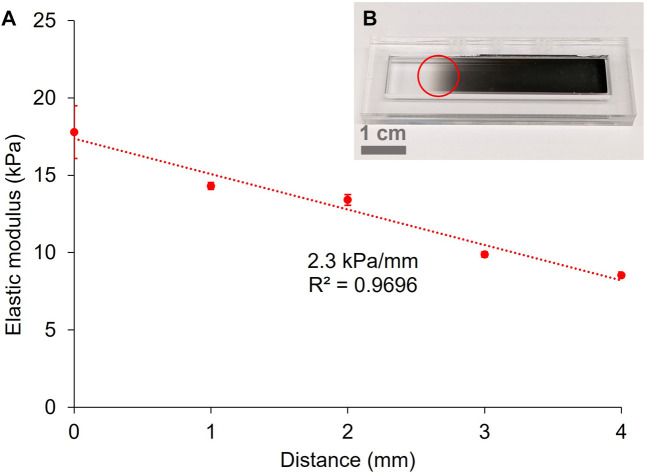
Gradient of stiffness on a 10 wt% GelMA hydrogel crosslinked for 4 min under the green LED **(A)** (error bars = standard deviation of the force maps). Photograph of the gradient filter (0.04–4.0 OD) used for the patterning of hydrogels **(B)**. The red circle represents the approximate position of the sample under the mask.

To improve the fidelity of the pattern, a commercially available optical filter was used to produce gradients. It consisted of a glass slide covered with a progressive metal coating that produces a continuous gradient of optical density (OD), from 0.04 to 4.0 over 50 mm (Figure 4B). The optical filter was placed on the mould on top of the pre-gel solution and then exposed to the 525 nm LED. For this experiment, an exposure time of 4 min was chosen. Figure 4A shows the elastic modulus of a 10 wt% GelMA hydrogel patterned with the filter positioned where the transition from transparent to opaque is visible to the naked eye (Figure 4B).

By probing the hydrogel along the axis aligned with the gradient filter, we measured a linear relationship between the elastic modulus and the distance across the hydrogel (corresponding to the increasing OD of the gradient filter), with a gradient of 2.3 kPa/mm (*R* ^2^ = 0.9696). According to Vincent et al. (2013), this stiffness gradient lies between a physiological gradient (around 1 kPa/mm) and pathological gradients (between 10 and 40 kPa/mm) for fibrotic tissue. As our goal is to achieve the modelling of pathological tissue presenting fibrosis, it is desirable to enable fabrication of steeper stiffness gradients.

### 3.4 Projection-Based Stiffness Patterning

**FIGURE 5:**
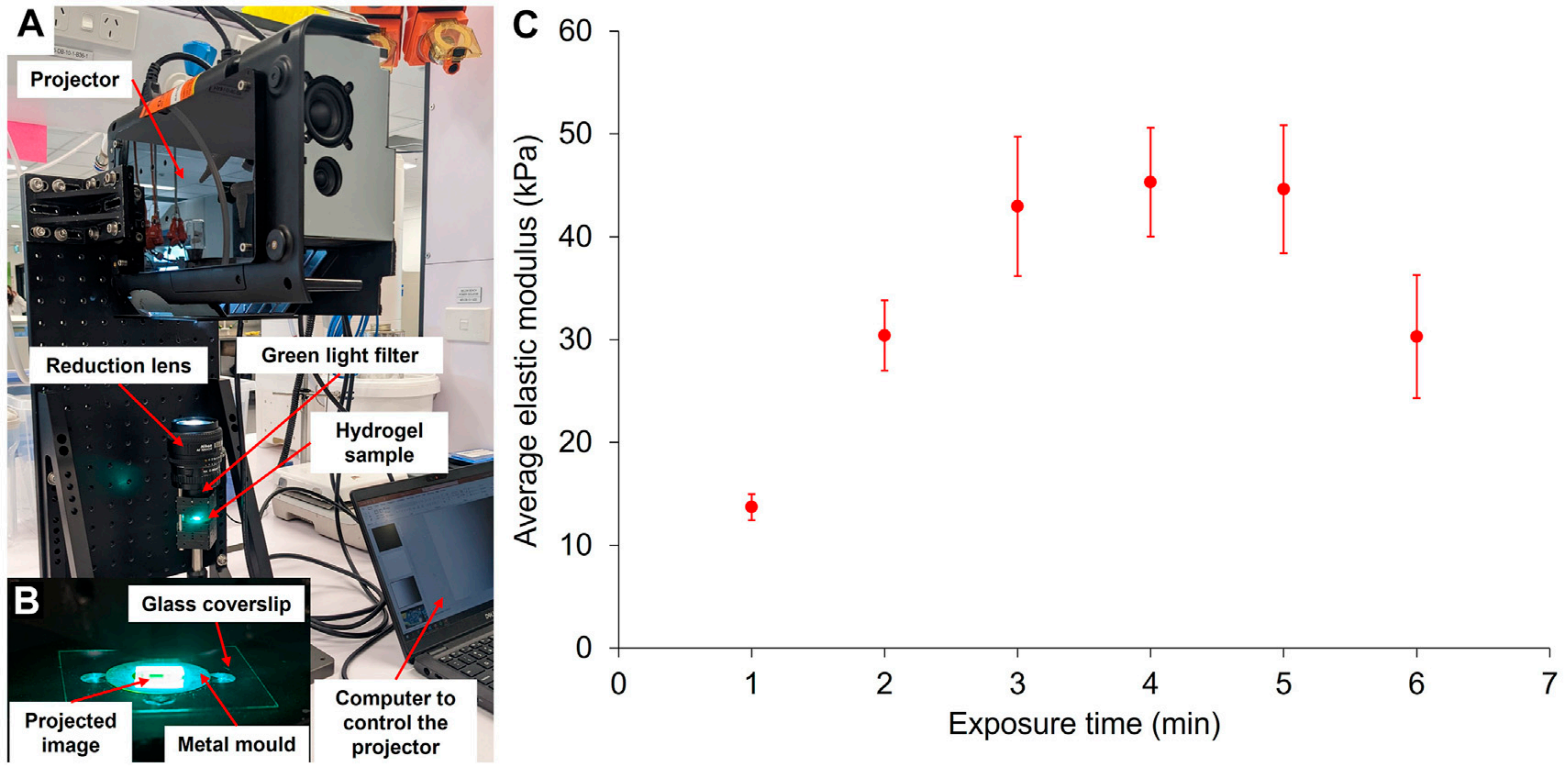
Crosslinking of GelMA via the projector system **(A)**. With this system, a digital image is directly projected at the surface of the pre-gel solution to create patterns of stiffness in the GelMA **(B)**. The resulting elastic modulus of 10 wt% GelMA hydrogels according to their exposure time under the projector has been measured by force indentation measurements with AFM **(C)** (errors bars: standard error over five force maps per exposure time).

A projection-based photopatterning system was developed to overcome the limitations of photopatterning using physical photomasks. This system consisted of a modified digital projector containing a digital micromirror device (DMD) which enabled direct control of irradiance across the gel, and the exposure time. This set-up was used to directly project the patterned light at the surface of the pre-gel solutions and thus cure the gels accordingly (Figures 5A,B). However, as with the green LED set-up, we first assessed the influence of exposure time to projected light at full irradiance (128 mW/cm^2^) on 10 wt% GelMA hydrogels’ elastic modulus, measured by force indentation measurements with AFM (Figure 5C).

Using the projector to cure the gels, we observed a similar trend as with the green LED, where the gel’s elastic modulus increased with increased exposure time until reaching a plateau and eventually decreased for longer exposure time, here 6 min. Again, this decrease in elastic modulus for longer exposure times may be due to photobleaching of the eosin Y. The plateau was reached for a shorter exposure time than the green LED, around 3 min. This phenomenon is somewhat unexpected since the irradiance of the projector is lower than the one of the green LED (160 mW/cm^2^). However, like with the LED, the maximum achievable elastic modulus remained at around 45 kPa, confirming that this is the limit for 10 wt% GelMA hydrogels with a DoF of 80%. The difference in temperature of the sample during light exposure between the two systems could also explain why similar moduli can be achieved with lower light intensity. Indeed, under the projector, the samples only heat up to a maximum of 22.5°C after 3 min of exposure, whereas in the case of the green LED, the temperature of the gel under the glass coverslip reaches 41.8°C after 3 min. As previously mentioned, a disorganisation of the gelatine network due to high temperature could explain the lower stiffness observed with the LED.

#### 3.4.1 Binary Pattern

The projector system was compared with the printed photomasks by fabricating binary gels with two areas of different greyscale values: one area with a value of 1 (white, meaning displaying the maximum irradiance of 128 mW/cm^2^) and one with values of 0.80 (74.2 mW/cm^2^), 0.67 (47.6 mW/cm^2^) or 0.5 (17.8 mW/cm^2^) (with 0 being black and displaying an irradiance of 1.0 mW/cm^2^). The relationship between the defined greyscale value in the software and the projected irradiance was determined with a photometer and is represented in Supplementary Figure S8. This relationship is non-linear, due to gamma corrections being applied in the software and the projector (Nesbitt et al., 1999).

**FIGURE 6:**
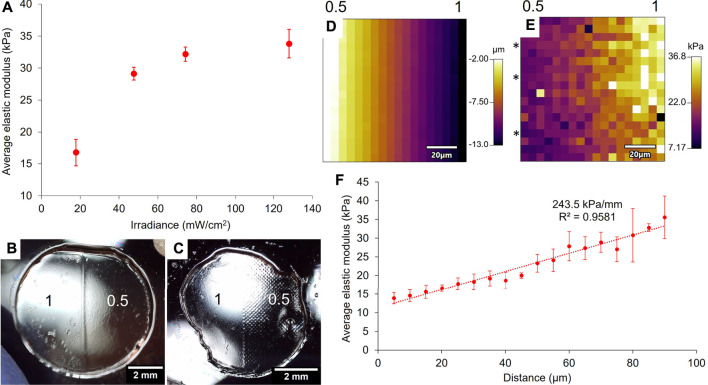
Relationship between the projected irradiance and the 10 wt% GelMA gels’ elastic moduli after exposure of 4 min **(A)**. The average elastic moduli have been assessed by force indentation measurements with AFM (error bars: standard error over at least four force maps per area). Images of the gels taken with a USB microscope show that the projector system does not produce sub-patterning over the masked area **(B)** compared to patterned gels prepared with the printed photomask **(C)** (scale bars = 2 mm). The numbers on **(B,C)** are the greyscale values that were applied to the gels. The boundary between the two areas of a 1/0.5 binary gel crosslinked for 3 min was probed with AFM and showed a height gradient **(D)** as well as a stiffness gradient on the force map **(E)** (scale bars: 20 μm). The numbers above maps **(D,E)** represent the orientation of the boundary in terms of greyscale values. Three lines on the force map **(E)** were averaged (lines identified with *) and plotted against the distance to show the strength of the gradient **(F)** (error bars: standard error of the mean over the three lines).

10 wt% GelMA binary hydrogels were crosslinked for 4 min under the projector and the mechanical properties of both areas were probed with force indentation measurements with AFM. Figure 6A shows the relationship between the projected irradiance and the resulting elastic modulus of the hydrogel. As with the printed photomasks, the modulation of irradiance induced the modulation of the GelMA hydrogels’ elastic moduli, in a non-linear fashion (Figure 3), saturating in this case at about 80 mW/cm^2^.

A significant improvement with the use of the projector is the absence of sub-patterning, as was observed with the printed photomasks. Under a USB microscope, we did not observe any sub-patterning on the surface of the gel made with the projector (Figure 6B), whereas the sub-patterning of the ink dots was clearly visible on the surface of the gel made with the printed photomask (Figure 6C). Indeed, the pixels projected at the surface of the hydrogel with the projector set-up were considerably smaller than the ink dots of the printed masks (around 120 μm): the native resolution of the projector is 1,024 × 768 pixels, and the projected image was measured to be approximately 8 mm × 6 mm, which gives a pixel size of approximately 8 μm. The hydrogel’s surface thus appears homogeneous at the microscopic level, and no sub-patterning was detected with the AFM either. We should also note that, contrary to the printed photomasks where the grey colour was made of black ink dots more or less spaced on the surface, the grey colour created by the projector is a modulation of the irradiance. This means that all the pixels have the same intensity within the grey area of the binary pattern, which is why no sub-patterning should be observed. The projector-based photopatterning therefore ensures a more homogeneous stiffness environment at the microscopic scale for the cells seeded into or onto hydrogels.

The boundary between the two areas indicates the maximum stiffness gradient that can be achieved with these crosslinking conditions. A force map of 90 μm × 90 μm was made at the interface between areas of a 10 wt% GelMA hydrogel exposed to greyscale values of 0.5 (17.8 mW/cm^2^) and 1 (128 mW/cm^2^) for 3 min. The force map gave the height profile (Figure 6D) of the area and its elastic modulus profile (Figure 6E), and from left to right, the transition goes from the area exposed to 17.8 mW/cm^2^ to the area exposed to 128 mW/cm^2^. Figure 6D shows that the left part of the map sits 11 μm higher than the right, and Figure 6E shows that the left part has a lower elastic modulus than the right. These results are consistent because if the gel displays a low elastic modulus, the gelatine network is loosely crosslinked, which means that it can take up more water and is thus more swollen (Oh et al., 2022). On the contrary, a higher elastic modulus means a more crosslinked network and thus a reduced swelling. Three lines of elastic modulus on the force map were averaged and plotted in Figure 6F. The average elastic modulus along this gradient ranged from 13.9 to 35.6 kPa over a length of 85 μm, which resulted in a slope of 243.5 kPa/mm. This type of very steep gradient is actually characteristic of tissue interfaces and not of stiffness gradients found in fibrotic tissues (Vincent et al., 2013). The average elastic modulus over each area of the binary gel was 4.8 kPa for the 0.5 area and 30.0 kPa for the 1 area. The fact that the value of 5 kPa was not reached on the force map shows that the boundary is even larger than 90 μm, even though on the projected image, the boundary is defined by a single line transition from a grey pixel to a white one. Diffraction of light and diffusion of radical species at the boundary can explain this result.

#### 3.4.2 Gradient Pattern

**FIGURE 7:**
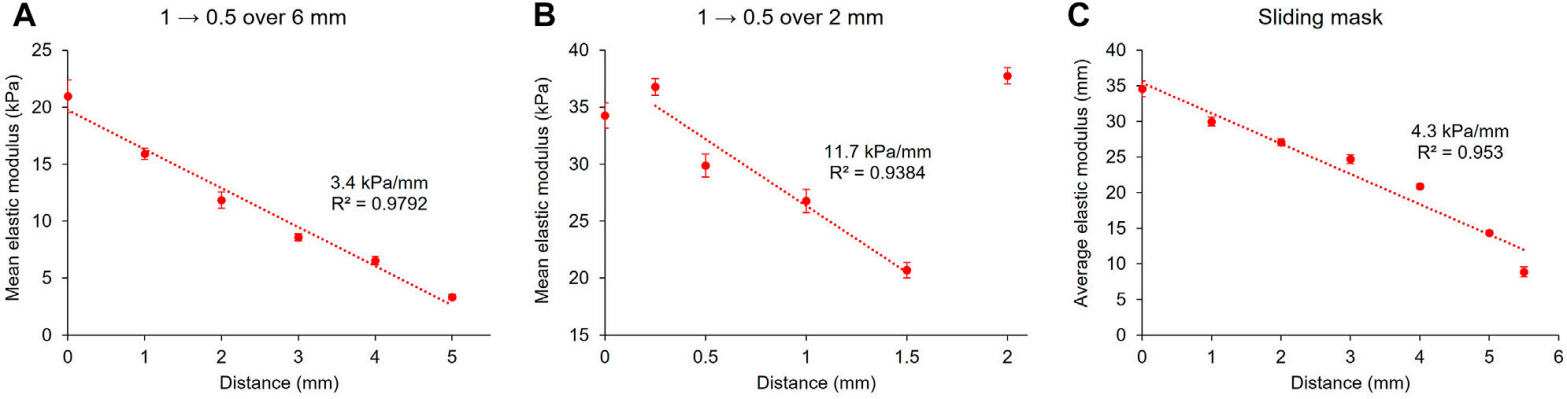
Stiffness gradients with the projector set-up. 10 wt% GelMA hydrogels were crosslinked for 4 min with a gradient ranging from greyscale values of 1 to 0.5 over a distance of approximately 6 mm **(A)** or 2 mm **(B)**. Another method to produce a stiffness gradient with a sliding mask was also used **(C)**. Error bars: standard deviation of the elastic modulus over the force maps.

Two methods were used to produce stiffness gradients with the projector. The first was to create greyscale gradients projected on the pre-gel solutions. Here again, several designs were made. A disk displaying a gradient with greyscales values from 1 to 0.5 over its diameter (6 mm) was first projected at the surface of the pre-gel solution for 4 min (Figure 7A). This resulted in a linear gradient (*R* ^2^ = 0.9792) of stiffness over the sample’s diameter, ranging from approximately 21–3.3 kPa, with a slope of 3.4 kPa/mm. This value remains in the range of physiological gradients for living tissues (Vincent et al., 2013). To increase the strength of the gradient with the same greyscale values, we reduced the distance over which the gradient spreads to 2 mm instead and exposed the pre-gel solution to this pattern for 4 min. Figure 7B shows that the fully crosslinked gel has an elastic modulus of approximately 35 kPa (the first two points on the left and the last on the right of the graph) and that the greyscale gradient induced a reduction of the elastic modulus to approximately 21 kPa. The slope of this gradient was 11.7 kPa/mm (*R* ^2^ = 0.9384), which is in the range of stiffness gradients for pathological tissues.

It was noted that the magnitude of the elastic moduli of the 6 and 2 mm gradients are not consistent for the same greyscale values. This can possibly be explained by the inherent mechanical variations encountered in our system between different samples. It should also be noted that diffusion of the reactive species at the boundaries of the 2 mm gradient may have induced a higher modulus because of the presence of the fully crosslinked surrounding gel.

It is important to note that the greyscale gradient projected by the projector system is not linear due to the gamma correction applied. Surprisingly, even though the greyscale gradients were not linear, the stiffness gradients were approximately linear, with *R* ^2^ > 0.93 in each case. As we saw in Figure 6A, the crosslinking phenomenon is itself non-linear, and when combined with the non-linearity of the greyscale gradient, it has resulted in an approximately linear gradient.

To avoid the non-linearity of gamma correction, we used a sliding mask to produce a stiffness gradient (Figure 7C). The principle of the sliding mask is that the pre-gel solution is progressively exposed to the light, from one extremity of the sample to the other. This results in different exposure times along the sliding direction of the mask and thus in various crosslinking densities and elastic moduli. If the speed of the sliding mask is constant throughout the length of the sample, this would have the same effect as a true linear greyscale gradient (as defined by a linear colour space): instead of controlling the projected irradiance, the exposure time is tuned along the sample’s diameter. Figure 7C shows a 10 wt% GelMA hydrogel cured by projecting onto it a video of a sliding mask. The mask took 1.5 min to cross the sample, which was then fully exposed to the light for one additional minute. The resulting gel exhibited an elastic modulus of approximately 35 kPa at one extremity to 9 kPa to the other, resulting in an overall slope of 4.3 kPa/mm. The linear regression to the stiffness gradient with this method produced a *R* ^2^ value of 0.95. However, upon inspection of the curve, a non-linear curve shape is indicated. This is in fact according to expectations due to the non-linear relationship between irradiance and stiffness in this system.

### 3.5 Complex Shape Designs

**FIGURE 8:**
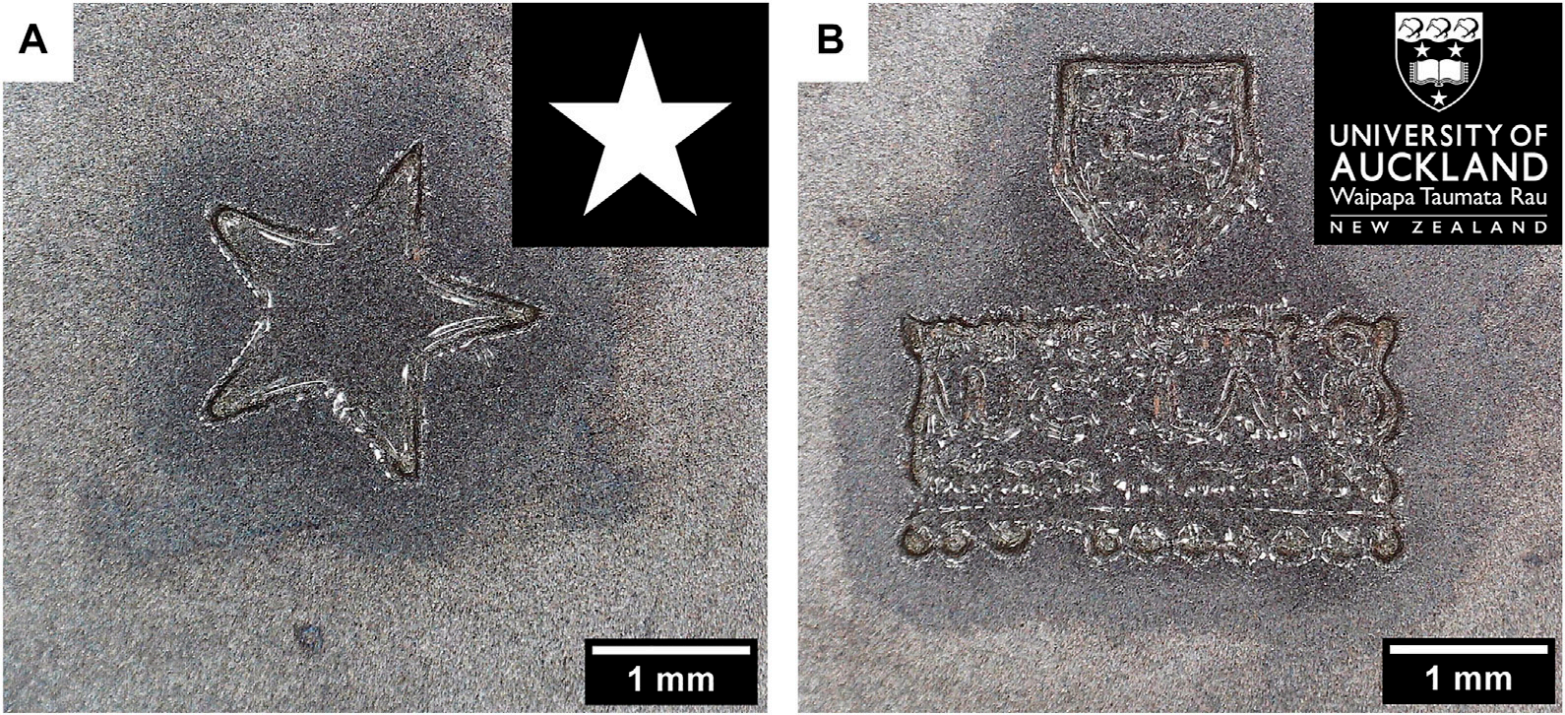
Complex shapes such as a star **(A)** or the University of Auckland logo **(B)** were projected for 4 min at the surface of a 10 wt% GelMA pre-gel (scale bars: 1 mm). It resulted in hydrogels with specific designs (the gels were dried for 1 h at RT before the pictures were taken).

Finally, we demonstrated the versatility of the projector set-up by fabricating complex shapes by projecting them on the surface of the pre-gel solution. Figure 8 shows examples of structures that can be fabricated by this method. Black colour was displayed around the white shape and prevented the pre-gel solution from crosslinking, thus leaving only the crosslinked shape after rinsing.

With the star shape, Figure 8A shows that the diffusion of the radical species potentially makes a less accurate design of the star, especially at the junctions of the branches. It is also worth noting that the gels have been dried at RT for 1 h before taking photos, as the water surrounding the gel hindered the precise observation of the shapes under the microscope. It is also possible that the shape inaccuracy was a result of drying.

Figure 8B shows how complex and fine these constructs can be. There is, however, a limit to the resolution of these details in the gel. For instance, the word “AUCKLAND” could be easily read on the gel, but for instance “NEW ZEALAND”, which had smaller dimensions on the projected picture, was not legible.

These results show that the projector set-up is a simple and versatile tool also able to produce hydrogels with specific shapes. Further development will still be needed to improve the fidelity of the constructs, by optimising the hydrogel composition for instance.

We report here the simple transformation of a commercially available projector containing a DMD to easily pattern and shape GelMA hydrogels with visible light. Such devices have already been used to pattern the stiffness of different types of hydrogels such as polyethylene glycol diacrylate (PEG-DA) by either crosslinking (Oh et al., 2022) or de-crosslinking the polymer network (Norris et al., 2016). Contrary to most studies that use UV light to crosslink and pattern GelMA (Major et al., 2019), we show here the feasibility of photopatterning GelMA with visible light via activation of eosin Y at 515 nm. Visible light has been employed to pattern DNA in a PEG-DA matrix with the help of photoinitiators sensitive to blue light (470 nm) (Dorsey et al., 2019), or to reversibly pattern with 450 nm light hydrogels made out of poly (hydroxyethyl acrylate) and a redox-responsive crosslinker (Amir et al., 2019). Several studies tried to pattern GelMA hydrogels using DMDs (Grogan et al., 2013; Wang et al., 2018, 2021), but this is, to our knowledge, the first example where the stiffness of GelMA has been patterned using projected visible light. Several studies have successfully 3D printed precise structures with GelMA hydrogels using visible light (Lim et al., 2018; Wang et al., 2018; Kumar et al., 2021; Levato et al., 2021), but without trying to control at the same time the stiffness of the scaffolds. In terms of achievable gradients, our system can generate stiffness gradients with slopes ranging from several hundreds of kPa/mm (at boundaries between areas of defined stiffness) to a few kPa/mm, which spans physiological and pathological gradients in tissues and at tissue interfaces (Vincent et al., 2013). Thus, GelMA hydrogels prepared by the method described here can be better suited for modelling fibrotic tissue *in vitro*, as well as for mechanotransduction studies to investigate the influence of matrix mechanical properties on stem cell behaviours, which also often use stiffness gradients on their scaffolds (Wang et al., 2012; Mosley et al., 2017; Chin et al., 2021). More generally, this method could also be adapted to other hydrogel systems that require light-based chemical crosslinking, such as methacrylated hyaluronic acid (Chen et al., 2021) or poly (ethylene glycol diacrylate) (PEGDA) (Nachlas et al., 2018), and that employ visible-light activated photoinitiators, which have been found to have a better light penetration depth in the gel compared to UV light, enabling thicker hydrogel constructs to be fabricated (Lim et al., 2019).

## 4 Conclusion

This work demonstrates the possibility of controlling and patterning the stiffness of GelMA with visible-light photo-crosslinking. Two set-ups were used for this purpose: one with a green high-power LED and physical photomasks, and another consisting of a projector mounted on an optical set-up to directly project patterns with green light at the surface of the samples. For both set-ups, the relationship between the hydrogel’s elastic modulus and the exposure time to the green light displayed an increase in stiffness with increasing exposure times. We also demonstrated that stiffness photopatterning was possible with both set-ups, in the form of binary hydrogels or gels presenting stiffness gradients. The projector system presented a much better fidelity of the patterns compared with photomasks and enabled the fabrication of constructs with complex shapes. Consequently, this study shows promising results for the fabrication of specific hydrogel designs in which cells can be safely encapsulated thanks to the use of visible light. With this method, GelMA hydrogels with well-defined stiffness patterning can then be employed to study mechanotransduction and cell fate in environments with different mechanical properties, which can potentially be used to build *in vitro* models of scar tissue and help finding novel fibrosis treatments.

## Data Availability

The original contributions presented in the study are included in the article/Supplementary Material, further inquiries can be directed to the corresponding authors.
