# Supplementary material for: Visible-Light Stiffness Patterning of GelMA Hydrogels Towards In Vitro Scar Tissue Models
Source: Front Cell Dev Biol. 2022 Jul 5;10:946754. doi: 10.3389/fcell.2022.946754 (PMC9294371; doi:10.3389/fcell.2022.946754)
Supplement: Supplementary file 1 [file DataSheet1.docx]

Supplementary Material

# Examples of Hertz model fits on force-indentation curves obtained with Atomic Force Microscopy (AFM)


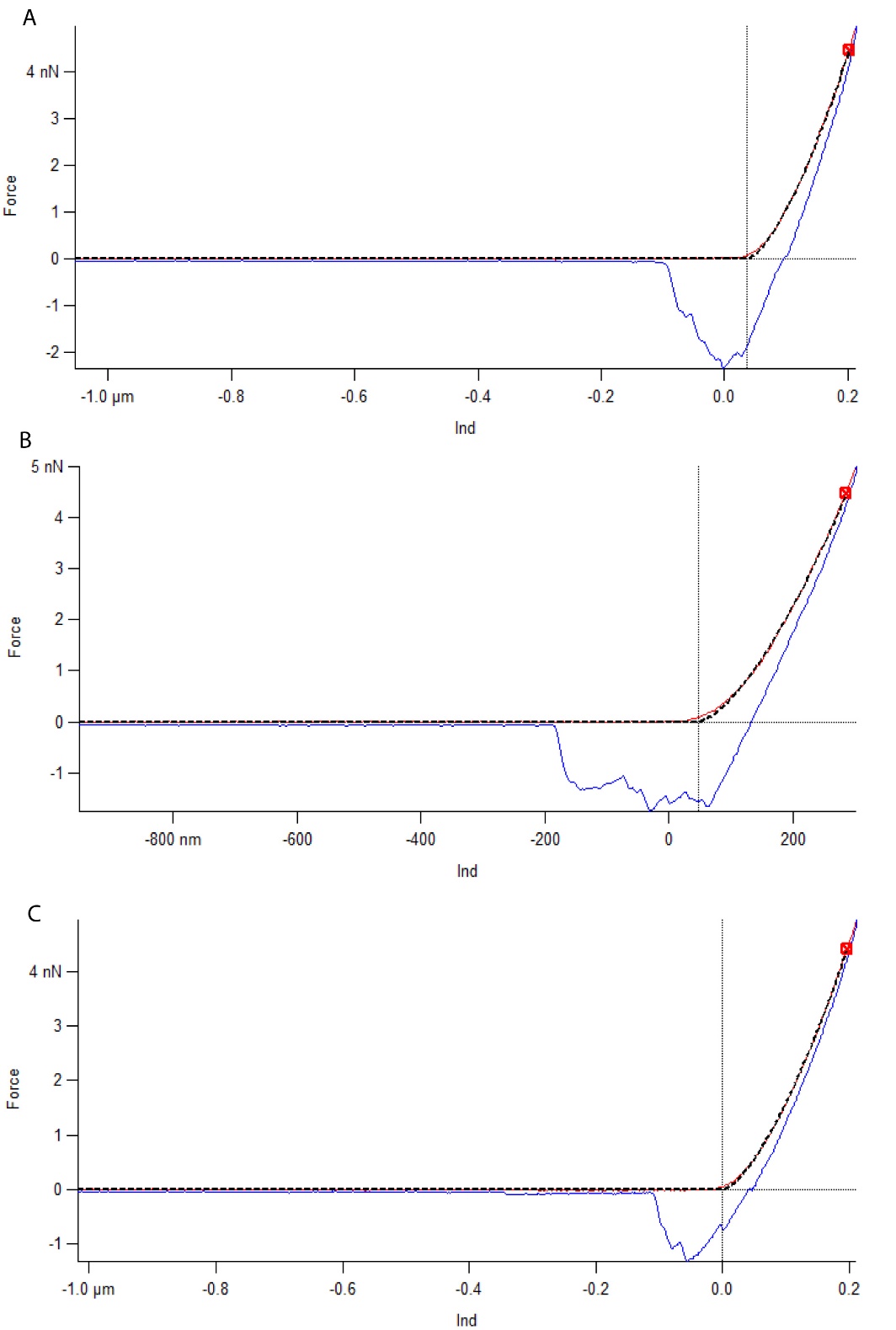


**Supplementary Figure S1.** Screenshot of the AR16 software to show examples of the Hertz fit (black dotted line) on the approach section (red line) of force-indentation curves obtained on different GelMA samples with AFM (blue line = retraction curve). (A) Force-indentation curve obtained on a 10 wt% GelMA hydrogel crosslinked for 4 min under the green LED. (B) Force-indentation curve obtained on a 10 wt% GelMA hydrogel exposed for 4 min to a 50 % opacity printed mask with the LED. (C) Force-indentation curve obtained on a 10 wt% GelMA hydrogel exposed for 4 min to a greyscale gradient from 0.5 to 1 over 2 mm with the projector (the probing position was at the soft end of the gradient).

# Absorbance spectrum of eosin Y


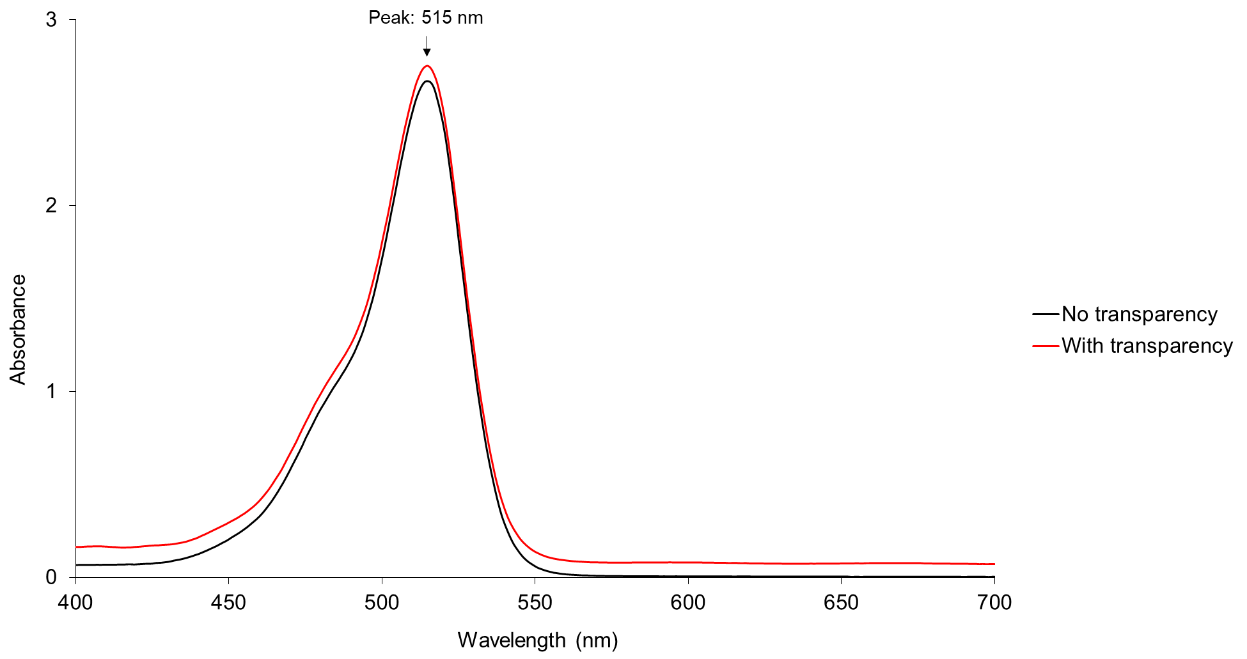


**Supplementary Figure S2.** Absorbance spectra of a solution of eosin Y in PBS measured with UV-vis spectroscopy, with and without a transparency sheet between the cuvette and the light source.

# Sample set variation for stiffness vs. exposure time experiments


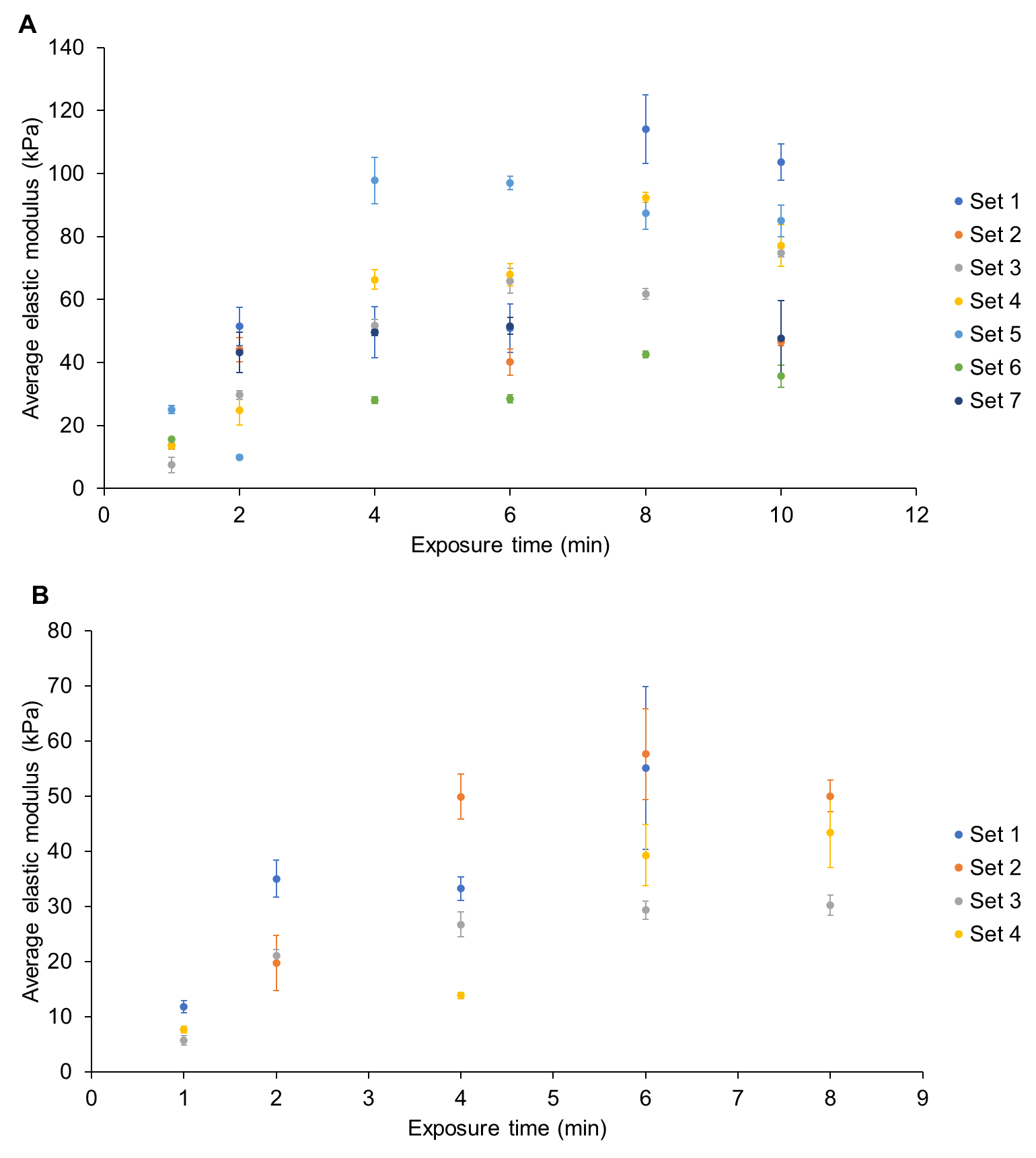


**Supplementary Figure S3.** Variation between different sample sets when probing the elastic moduli of hydrogels according to the exposure time to the green LED for 15 wt% GelMA hydrogels (A) and 10 wt% GelMA hydrogels (B).

# Temperature over time under the high-power LED and the projector


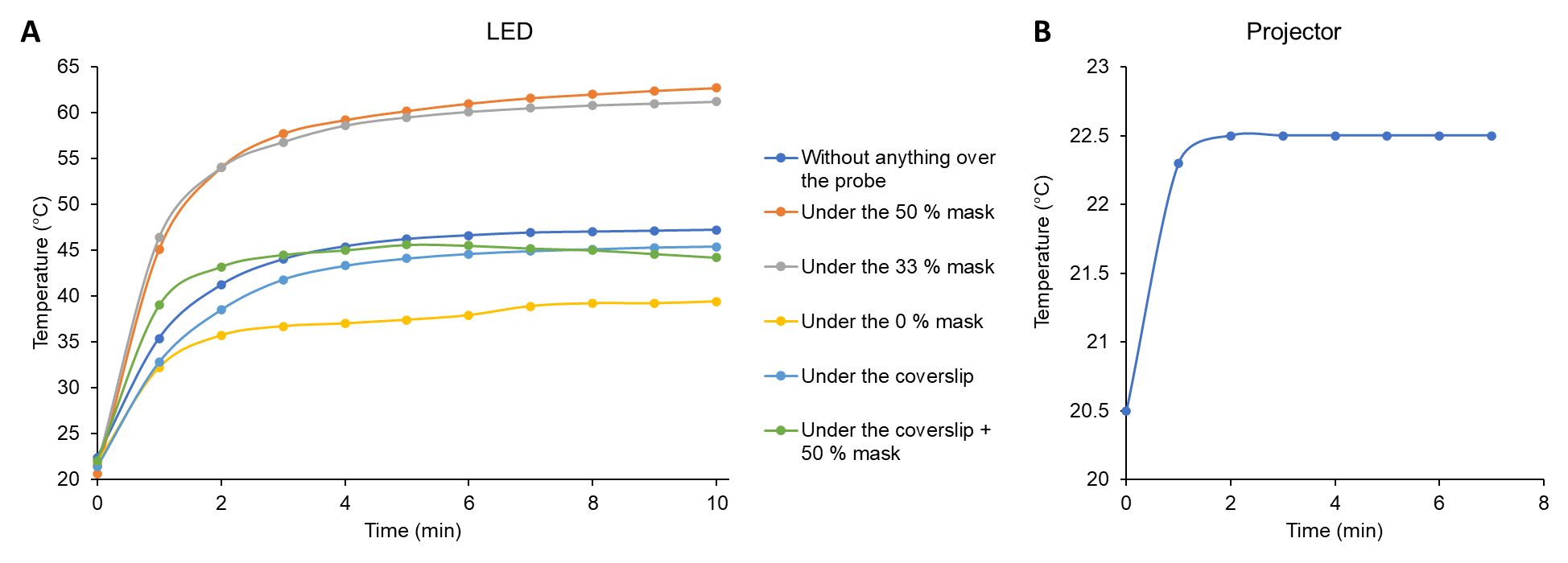


**Supplementary Figure S4.** Variation of the temperature over time under the high-power green LED at full irradiance (160 mW/cm^2^) under different conditions (A), and under the projector at full irradiance (128 mW/cm^2^), below a glass coverslip (B).

# Influence of pre-curing physical crosslinking on the hydrogels’ mechanical properties


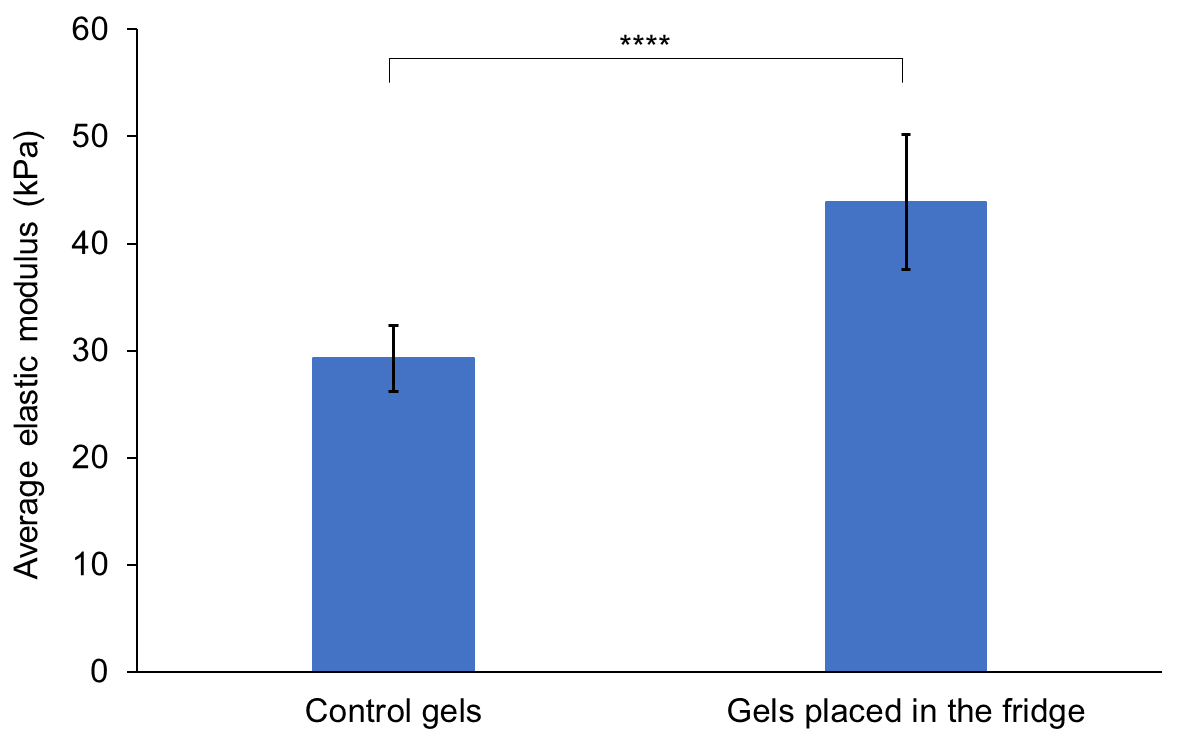


**Supplementary Figure S5.** AFM measurements of the average elastic modulus of 10 wt% GelMA hydrogels kept at 4 °C for 15 min before being crosslinked for 4 min at RT, compared to hydrogels that were directly crosslinked for 4 min at RT after pipetted in the mould (error bars: standard error, N = 9 force maps for control gels, N = 18 force maps for gels placed in the fridge, p < 0.0005).

# Transmittance of the printed photomasks measured by UV-vis spectroscopy

| **Printed photomask opacity (%)** | **Relative measured transmittance (%)** | **Calculated irradiance for the sample under the 525 nm LED (mW/cm^2^)** |
| --- | --- | --- |
| No mask | 100 | 160 |
| 0 | 83.5 | 134 |
| 33 | 17.6 | 28.1 |
| 50 | 4.70 | 7.51 |
| 100 | 1.80 | 2.88 |

**Supplementary Table S6.** Resulting irradiance of the green LED according to the printed photomask opacity after transmittance measurements with UV-vis spectroscopy.

# Sub-patterning of the hydrogels made with printed photomasks


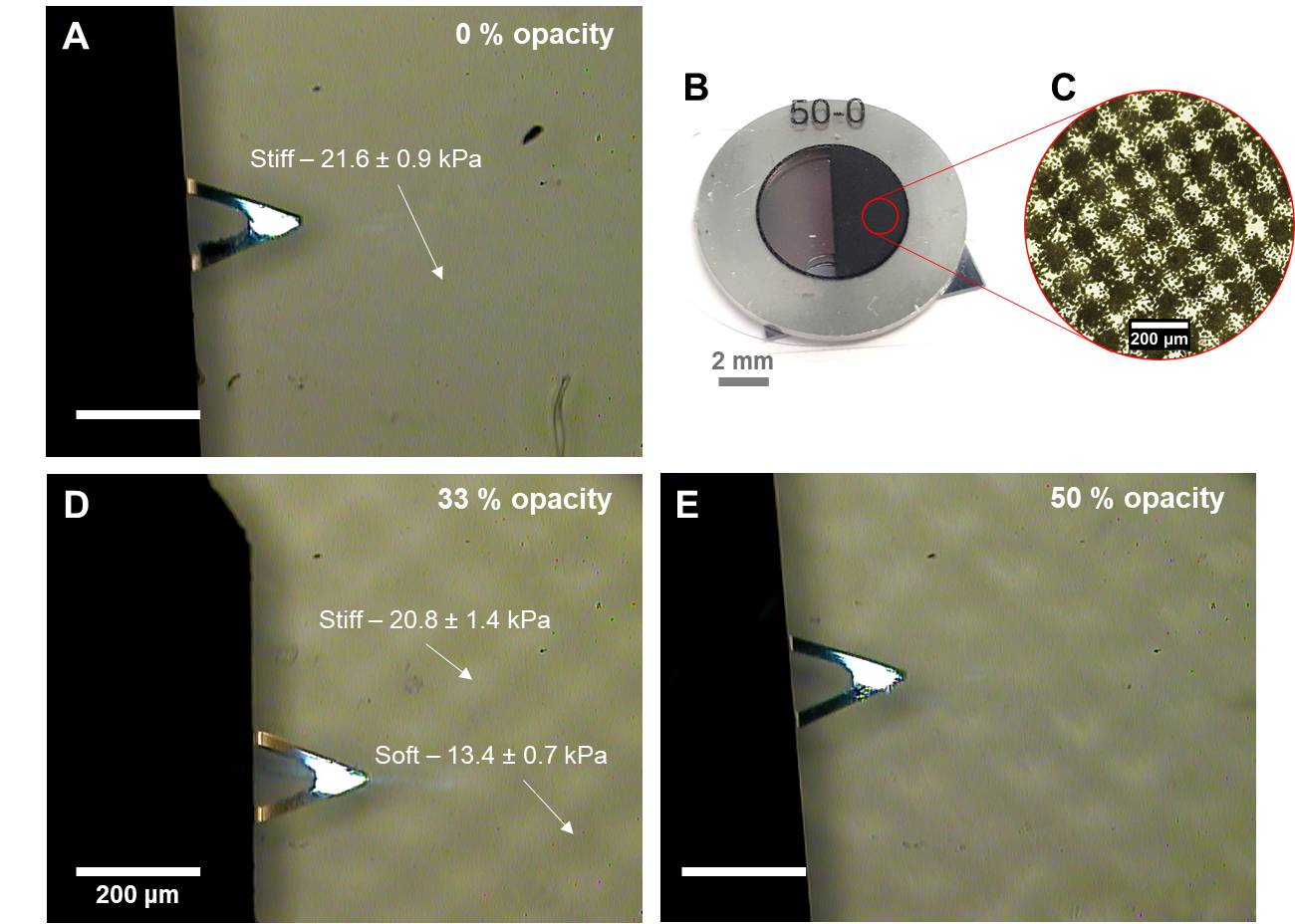


**Supplementary Figure S7.** Sub-patterning observed on binary gels under the AFM objective. The printed part of the photomask (B) presents an inkblot distribution with transparent holes in-between (C, scale bar: 200 μm), which leaves a pattern at the surface of the hydrogel that was below the opaque zone of the photomask (D for 33 % opacity and E for 50 % opacity mask). As a comparison, the transparent side of the photomask does not induce any sub-patterning at the surface of the hydrogel (A). When probing the different zones at the hydrogel’s surface, a significant difference in elastic modulus is observed and confirms the sub-patterning. Scale bar on pictures A, D and E: 200 μm.

# Relationship between greyscale value and irradiance with the projector set-up


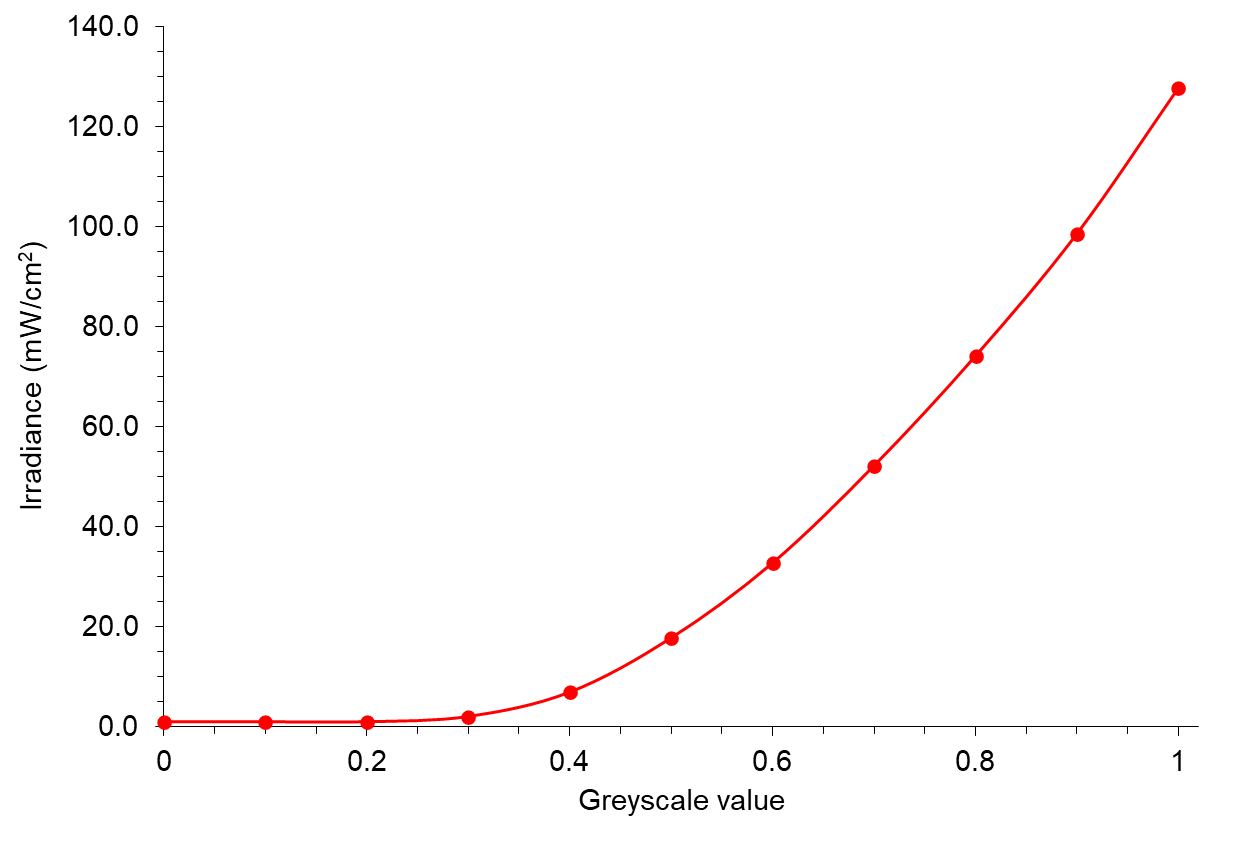


**Supplementary Figure S8.** Measured irradiance of the projector according to the defined greyscale value in the presentation program.
